# Successful treatment of rheumatoid neutrophilic panniculitis with tofacitinib^☆^

**DOI:** 10.1016/j.abd.2023.05.010

**Published:** 2024-04-23

**Authors:** Hiram Larangeira de Almeida Junior, Vitor Dias Furtado, Viviane Siena Issaacson, Ana Letícia Boff

**Affiliations:** a Postgraduate Degree in Health and Behavior, Universidade Católica de Pelotas, Pelotas, RS, Brazil; b Department of Dermatology, Universidade Federal de Pelotas, Pelotas, RS, Brazil; c Faculty of Medicine, Universidade Federal de Pelotas, Pelotas, RS, Brazil; d Private Practice, Pelotas, RS, Brazil; e Santa Casa de Misericórdia de Porto Alegre, Porto Alegre, RS, Brazil

Dear Editor,

The Janus kinase (JAK) family of enzymes, consisting of proteins JAK1, JAK2, JAK3, and TYK2, is a group of tyrosine kinases of great importance in the intracellular signalization, which, when activated by cytokines and growth factors, act on the signal transducers and activators of transcription (STAT), allowing their dimerization and translocation to the nucleus, regulating gene expression and transcription.^1^ This signaling pathway, called JAK/STAT, is related to several biological functions, such as proliferation, apoptosis, differentiation, immune regulation, and also hematopoiesis.^2^

JAK inhibitors (JAKi) are promising in the treatment of several rheumatological, hematological, and dermatological diseases. JAKi are small molecules capable of blocking the binding of JAK to its intracellular domain of cytokine receptors, preventing its phosphorylation and STAT dimerization, abolishing signal transduction via JAK/STAT and, consequently, inhibiting the pro-inflammatory response.^3^

Different JAKs can be blocked selectively or together, allowing the development of targeted treatments with fewer adverse reactions. The first JAKi approved for the treatment of autoimmune diseases was Tofacitinib, a small synthetic molecule that targets JAK1 and JAK3, with lesser action on JAK2 and TYK2. The utilization of JAKi in inflammatory and autoimmune diseases has increased over the years, used in the treatment of rheumatoid arthritis, psoriatic arthritis, systemic lupus erythematosus, dermatomyositis, ulcerative colitis, myelofibrosis, polycythemia vera, alopecia areata, vitiligo and atopic dermatitis.^4^

A 60 year-old female patient with a history of seropositive rheumatoid arthritis (RA) was evaluated. She used several medication regimens and only achieved good disease control with the following regimens: etanercept, sulfasalazine and hydroxychloroquine. This treatment controlled the disease and was used for three consecutive years.

In the first half of 2020, she developed RA activity and worsening of the disease, with arthritis of the hands and wrists, and in the middle of that year she had the first cutaneous manifestations (extra-articular disease), characterized by bilateral painful nodules on the legs, some with skin surface elevation, other flat, and the clinical hypothesis of erythema nodosum was made (Fig. 1A).

**Figure 1:**
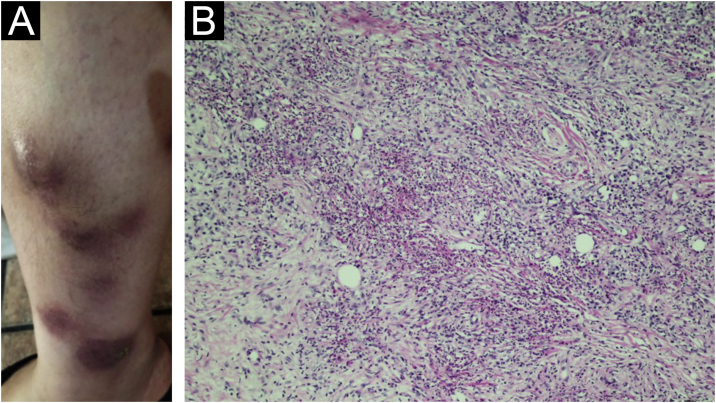
(A) Multiple erythematous nodules on the right leg at the onset of the presentation. (B) Neutrophil-rich infiltrate forming small abscesses in the dermis and subcutaneous tissue (Hematoxylin & eosin, ×200).

The skin lesions gradually worsened, some progressing to ulceration, while others showed infiltration only, they were extremely painful, and the total of 12 lesions had a high impact on her quality of life. Cultures for fungi and mycobacteria from lesion exudate were negative on two occasions.

Two nodular lesions were biopsied and histopathology showed an infiltrate rich in neutrophils forming small diffuse abscesses in the dermis and subcutaneous tissue (Fig. 1B). Neutrophilic pustules were seen in the papillary dermis (Fig. 2A) as well as vasculitis with fibrinoid necrosis of the vessel wall topped by neutrophils (Fig. 2B). Special stains for fungi and micobacteria were negative, so the findings were compatible with rheumatoid neutrophilic panniculitis.

**Figure 2:**
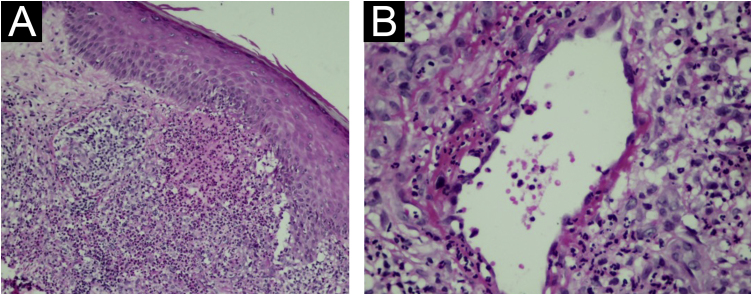
(A) Neutrophil accumulation in the papillary dermis (arrows; Hematoxylin & eosin, ×200). (B) Vasculitis with fibrinoid necrosis of the wall and neutrophils (Hematoxylin & eosin, ×400).

The patient medication regimen was changed several times after this worsening with extra-articular manifestations. She used the following at therapeutic doses: prednisone, abatacept, colchicine, hydroxychloroquine, methotrexate, and golimumab; all without joint or skin response.

Six months ago, the therapeutic regimen was changed to tofacitinib 10 mg per day, as monotherapy, and there was control of the joint disease and cutaneous manifestations (Figure 3, Figure 4), with complete healing of the lesions within 120 days. The painful symptoms completely disappeared.

**Figure 3:**
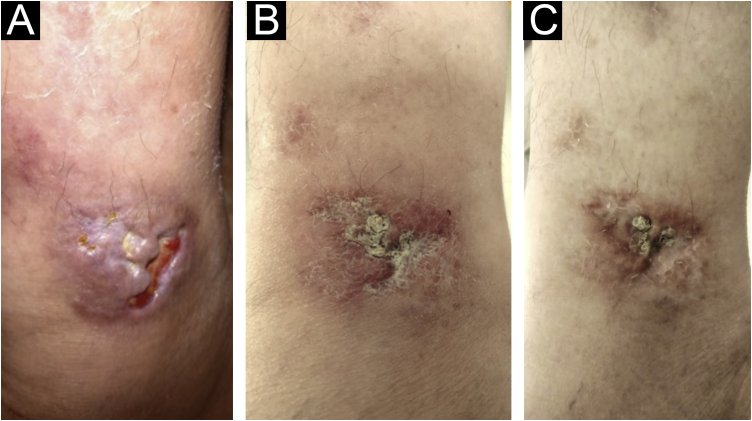
Therapeutic response. (A) Lesion on the right internal malleolus with infiltration, ulceration and necrosis. (B) After 60 days of treatment, showing significant reduction in infiltration and ulceration. (C) Complete resolution after 120 days.

**Figure 4:**
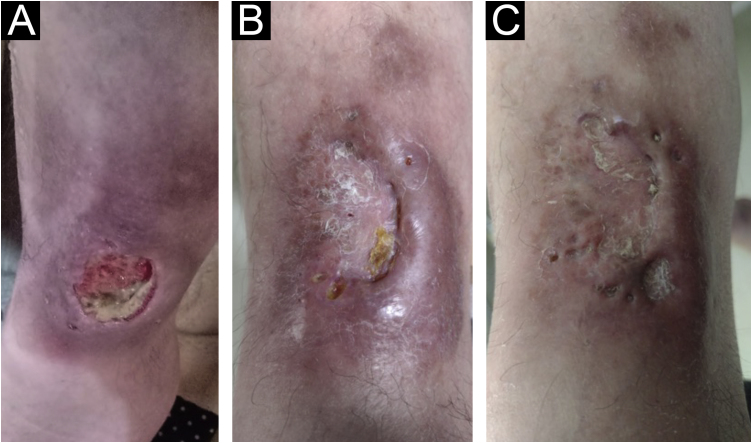
Therapeutic response. (A) Lesion on the left external malleolus with ulceration and infiltrated edges. (B) After 60 days of treatment, the ulceration resolved, but part of the infiltrated edge persisting. (C) Complete resolution after 120 days.

Neutrophilic panniculitis associated with rheumatoid arthritis (RA) is a condition described in 1988,^5^ and is less frequent than erythema nodosum associated with this rheumatological disease.^6^ It appears in long-term disease, with frequent involvement of the posterior surface of the legs and with ulceration,^5^, ^6^ similar to the present patient. This helps to differentiate it from erythema nodosum. On histopathology, there is a neutrophilic infiltrate in the hypodermis, which may be accompanied by vasculitis,^6^ as in the case described herein.

In a publication on the histopathological spectrum of skin lesions in RA, Magro et al. described three patterns: palisade granulomas, interstitial neutrophilia, and vasculopathy (lymphocytic, neutrophilic or granulomatous).^7^ Intersections between these patterns are possible, as in the present case, with dermal and hypodermal neutrophilia, and vasculopathy.

The clinical picture described in the present patient is very rare and should be known to dermatologists, as part of the spectrum of rheumatoid neutrophilic dermatoses.^8^ Moreover, resistance to several classic RA treatments and the excellent response to tofacitinib demonstrate new uses for this emerging treatment modality, for which there are also reports of successful use in other neutrophilic dermatoses, such as pyoderma gangrenosum.^9^

## Financial support

None declared.

## Authors’ contributions

Hiram Larangeira de Almeida Jr: Approval of the final version of the manuscript; design and planning of the study; drafting and editing of the manuscript; collection, analysis and interpretation of data; effective participation in research orientation; intellectual participation in the propaedeutic and/or therapeutic conduct of the studied cases; critical review of the literature; critical review of the manuscript.

Vitor Dias Furtado: Approval of the final version of the manuscript; design and planning of the study; drafting and editing of the manuscript; collection, analysis and interpretation of data; intellectual participation in the propaedeutic and/or therapeutic conduct of the studied cases; critical review of the literature; critical review of the manuscript.

Viviane Siena Issaacson: Approval of the final version of the manuscript; design and planning of the study; drafting and editing of the manuscript; collection, analysis and interpretation of data; intellectual participation in the propaedeutic and/or therapeutic conduct of the studied cases; critical review of the literature; critical review of the manuscript.

Ana Letícia Boff: Approval of the final version of the manuscript; design and planning of the study; drafting and editing of the manuscript; collection, analysis and interpretation of data; intellectual participation in the propaedeutic and/or therapeutic conduct of the studied cases; critical review of the literature; critical review of the manuscript.

## Conflicts of interest

None declared.
